# Visual Acuity Provides a More Meaningful Measure of Vision-Related Functioning Than Mesopic Microperimetry in Age-Related Macular Degeneration Patients: A Cross-Sectional Study

**DOI:** 10.1167/tvst.15.2.5

**Published:** 2026-02-04

**Authors:** Francesco Cinque, Jeroen A. A. H. Pas, Anita de Breuk, Tom Heesterbeek, Caroline C. W. Klaver, Carel B. Hoyng, Yara T. E. Lechanteur, Caroline M. van Heugten

**Affiliations:** 1 Department of Ophthalmology, Donders Institute for Brain, Cognition and Behaviour, Radboud University Medical Center, Nijmegen, the Netherlands; 2 Department of Ophthalmology, Erasmus Medical Center, Rotterdam, the Netherlands; 3 Department of Epidemiology, Erasmus Medical Center, Rotterdam, the Netherlands; 4 Institute of Molecular and Clinical Ophthalmology, Basel, Switzerland; 5 Department of Neuropsychology and Psychopharmacology, Maastricht University, Maastricht, the Netherlands; Limburg Brain Injury Center, Maastricht University, Maastricht, the Netherlands

**Keywords:** visual acuity, microperimetry, clinical trials, age-related macular degeneration, patient-reported outcomes

## Abstract

**Purpose:**

Mesopic microperimetry is a promising tool to evaluate retinal function in clinical trials. Although visual function (VF), the ability to perform vision-related tasks, relates strongly to visual acuity (VA) in patients with age-related macular degeneration, the relationship between mesopic microperimetry and VF remains unclear.

**Methods:**

A cross-sectional study in patients with age-related macular degeneration was performed. VF was measured by questionnaire using a subset of the National Eye Institute 25-Item Visual Function Questionnaire. The macular integrity assessment microperimeter, was used with a 4–2 staircase strategy with a 10° diameter circular grid containing 37 loci. Three interpretations of the retinal sensitivity data were calculated: the mean of the 37 thresholds (mean sensitivity [MS]), the percent-reduced threshold (PRT), and the log-transformed candela mean (MS cd log). MS, PRT, and MS cd log were tested via stepwise hierarchical linear regression and *R*^2^.

**Results:**

We analyzed data from 102 patients (64 females [61%]; mean age, 71.8 ± 11.2 years). VF was explained best by VA (MS; *R*^2^ VA = 0.38, MS cd log; *R*^2^ VA = 0.38, PRT; *R*^2^ VA = 0.39). All retinal sensitivities contributed significantly to total *R*^2^. In the MS cd log model (total adjusted *R*^2^ = 0.52) the combined contribution of variance explained by VA and MS cd log was 46% (partial adjusted *R*^2^ = 0.46).

**Conclusions:**

Mesopic microperimetry is associated with VF but VA provides a more meaningful estimation of this same construct. These results suggest that VA provides stronger evidence of clinical efficacy.

**Translational Relevance:**

This study relates functional tests to daily vision-related functioning.

## Introduction

Age-related macular degeneration (AMD) is a progressive retinal disease that is currently the leading cause of irreversible vision loss in the world for elderly people.^1^ In early AMD, patients may suffer mild visual disturbances but the disease can progress to late-stage AMD, characterized by geographic atrophy (GA) or choroidal neovascularization, significantly reducing quality of life (QOL).^2^^,^^3^

Current trials in AMD use best-corrected visual acuity (BCVA) as a functional end point.^4^ However, BCVA, whether primary or secondary, has limitations in AMD.^5^ BCVA shows little change over the course of most trial durations because it is inadequately sensitive to changes in early, intermediate AMD, and extrafoveal GA; and may fail to sufficiently discriminate in late AMD with foveal involvement.^5^^,^^6^ For instance, BCVA cannot measure relevant extrafoveal improvement in (exudative) AMD.^7^ In GA, the slow development of foveal involvement might exceed the standard duration of most clinical trials.^8^^,^^9^ These shortcomings may negatively impact the measurability of a potential therapeutic

Mesopic microperimetry (MMP) is a promising psychophysical functional test that has already been implemented as a secondary outcome measure in GA clinical trials.^10^^–^^14^ MMP entails controlled retinal sensitivity mapping of the macula and simultaneous measurement of fixation stability.^6^ Patients are presented light stimuli of varying intensity in candela/m^2^, usually expressed in decibel (dB). The threshold, or retinal sensitivity, at which the patient can perceive this stimulus is then determined. BCVA is the ability to distinguish separate points at a distance over a high-contrast background, in a high luminance environment, exclusively reflecting foveal function, while MMP measures retinal function beyond the fovea.^15^^,^^16^ MMP might be used in future trials because of its discriminatory ability in earlier stages of AMD.^17^^,^^18^

An important argument to justify use of MMP as a primary end point would be to show that MMP is related to vision-dependent tasks or visual function (VF).^19^ VF is a measure of everyday vision-dependent tasks such as reading and perceiving facial expressions.^20^ Although VF relates strongly to VA in AMD patients, the relationship between MMP and VF remains unclear.^21^

This study investigates how much VF is explained by MMP and VA. The output of the MMP can be interpreted using various methods, including the geometric mean, which is the average of all thresholds in dB, a percentage score of loci scoring below threshold, and the novel approach of averaging the candela/m^2^ scores before log transformation. Because MMP output yields different variables, we aimed to explore these three distinct interpretations of retinal sensitivity in addition to fixation metrics in this study.

## Methods

### Study Design and Participants

This secondary baseline analysis was performed with data collected from January 2018 through April 2019 at the Radboud University Medical Centre in Nijmegen (the Netherlands) as part of the European Genetic Database (EUGENDA). All patients had at least one eye with macular drusen secondary to AMD. The exclusion criterion was incorrect alignment of the microperimetry grid on the fovea to ensure valid measurement. Two cohorts were defined based on VA: the best-seeing eye cohort and the worse-seeing eye cohort. This study was conducted in accordance with the tenets of the Declaration of Helsinki and was approved by the local ethics committee (2027–3535). All participants provided written informed consent before participating.

### Study Protocol

Presenting VA was measured using the Early Treatment Diabetic Retinopathy fast method.^22^^–^^24^ A printout of the Dutch consensus translation of the National Eye Institute 25-Item Visual Function Questionnaire (NEI-VFQ25)^25^ was handed out on site and completed using both eyes and reading glasses if necessary. MMP was performed with the macular integrity assessment microperimeter (CentreVue, Padova, Italy) using a 4–2 staircase strategy with a grid of 10° diameter containing 37 radially oriented points centered on the fovea. Trained personnel explained the procedure and performed a mock examination before actual examination at baseline to prevent a learning curve effect. MMP was not performed in mydriasis because the macular integrity assessment microperimeter is able to operate with a pupil diameter of 2.5 mm.^6^^,^^26^ The following imaging modalities were performed: color fundus photography (DRI Triton; Topcon, Tokyo, Japan), spectral-domain optical coherence tomography, and fundus autofluorescence (Spectralis HRA+OCT; Heidelberg Engineering, Heidelberg, Germany). All imaging was done in mydriasis. Ophthalmic history and medical history was assessed through questionnaires.

### Main Outcome: VF

The Long-Form Visual Functioning Scale (LFVFS-39) measuring VF was used.^27^ A LFVS-39 score of 100 indicates no difficulty in performing activities owing to vision, and 0 indicates a complete inability to perform activities owing to vision.^27^ The LFVFS-39 averages the recoded responses to items 2, 5, 6, 7, 8, 9, 10, 12, 14, A3, A4, A5, A6, A7, and, A8 from the NEI-VFQ. Despite the LFVFS-39 still retaining differential item functioning, that is, some items measure differently for sex,^27^ its primary advantage is its better precision over the LFVFS-25, which is comparatively smaller scale using solely the NEI-VFQ25. Application of this Rasch-based revision has since been manyfold.^21^^,^^28^

VF can be derived from the NEI-VFQ, the most used vision-related QOL (VRQoL) questionnaire in ophthalmology.^29^ Psychometric evaluation of the NEI-VFQ via Rasch analysis has shown that the NEI-VFQ violates unidimensionality; that is, it does not measure a single construct, which is not uncommon in QOL measurements.^27^ This can be resolved by segregating a subset of the original questions into two distinct measures of VRQoL: VF and socioemotional functioning.^27^ VRQoL is a multifaceted (ie, multidimensional) construct that includes extra domains such as emotional well-being, social participation, and economic considerations.^20^ VF and VRQoL are similar in that they can be measured through psychometric instruments.^20^^,^^29^^,^^30^ Although VRQoL is a valuable measure, its multidimensionality renders it more challenging to interpret because it cannot be represented by a single scale.^20^^,^^29^

### MMP Variables

**Figure 1.**
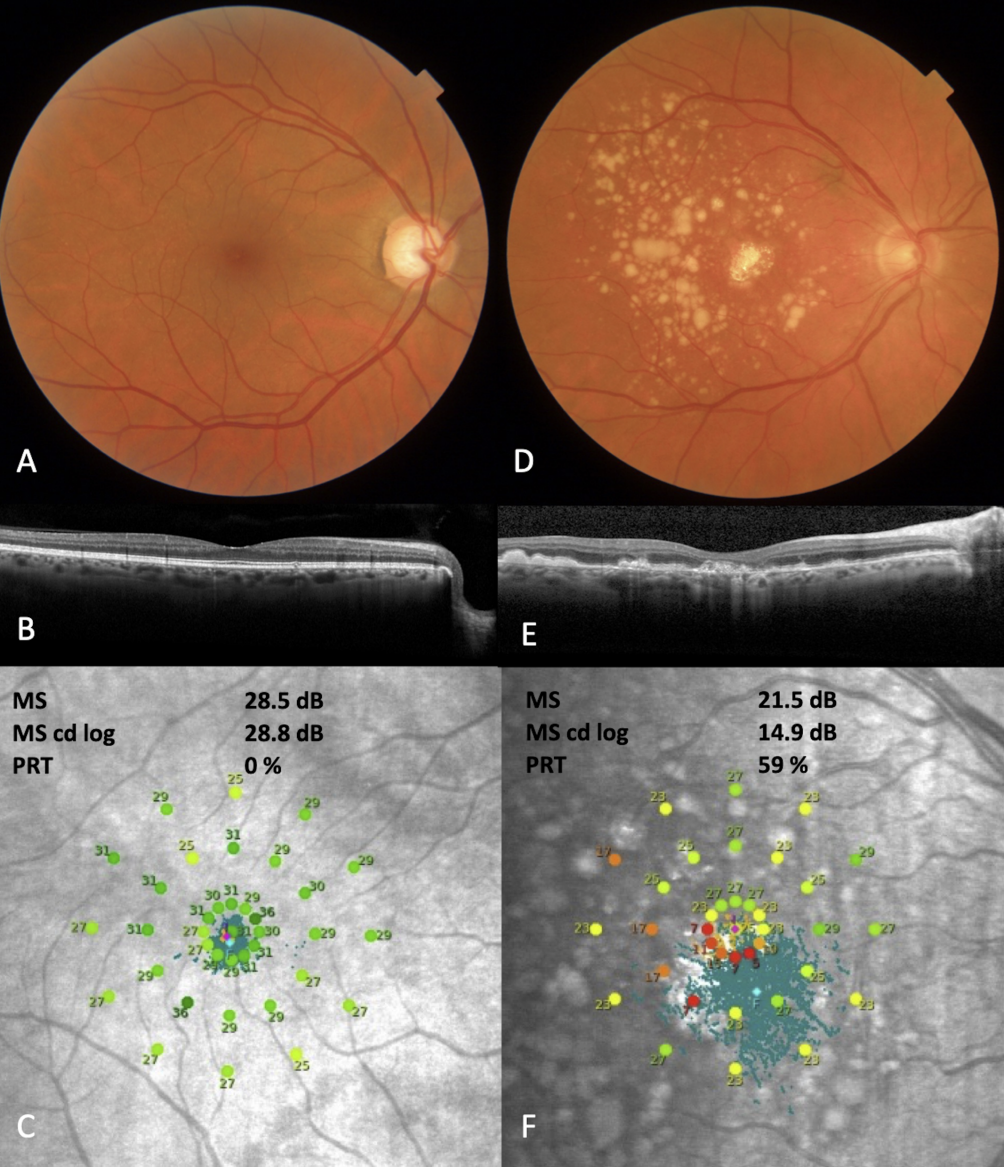
Example of retinal sensitivity values for early AMD Eye (*left*) and late AMD Eye (*right*). (**A**) Color fundus photography (CFP) of eye with early AMD. (**B**) Corresponding spectral-domain optical coherence tomography (SD-OCT) B-scan showing small drusen. (**C**) Corresponding retinal sensitivity. (**D**) Eye with advanced AMD. (**E**) Corresponding SD-OCT with hypertransmission and large drusen. (**F**) Corresponding retinal sensitivity. In (**C**) and (**F**), we show the MS, MS cd log, and PRT values. This figure demonstrates how different calculations of retinal sensitivity diverge in patients with varying stages of AMD.

Retinal sensitivity is measured at each of the 37 loci on a 0 to 36 dB scale such that 0 dB corresponds with the brightest stimulus at 318 cd/m^2^.^6^ Retinal sensitivity data can be analyzed differently.^5^^,^^6^ We explored three different strategies:

1) Mean sensitivity (MS); this is the average of all 37 thresholds in dB.
2) MS candela log (MS cd log). MS cd log is calculated by first converting each threshold into its corresponding candela/m^2^ value.^6^ Next, all thresholds in candela/m^2^ are averaged. Subsequently, all values are transformed back to a dB value.
3) Percent-reduced threshold (PRT); the number of thresholds below 25 dB divided by 37, expressed as a decimal value^5^^,^^6^ (Fig. 1).

Fixation metrics are provided in the form of P1, P2, BCEA-63, and BCEA-95.^31^ In brief, P1 and P2 describe the percentage of fixation points within 1° and 2° radii circles. BCEA-63 and BCEA-95, or bivariate contour ellipse area, describes the area in degrees (°) which contain 63% or 95% of fixation points.^31^

### Grading

Grading of color fundus photography, spectral-domain optical coherence tomography, and fundus autofluorescence was performed by experienced graders of the Eye-NED Reading Center using the modified version of the Wisconsin Age-Related Maculopathy Grading System.^32^ This was then reclassified into the Rotterdam Study (RS) classification.^33^ Patient stage is defined by the highest staged eye.

### Statistical Analysis

Exploratory simple linear regression of VF (LFVFS-39) was performed with the following variables measured in the best-seeing eye and worse-seeing eye respectively; MS (dB), MS cd log (dB), PRT (decimal value), P1 (%), P2 (%), BCEA-63 (°), and BCEA-95 (°). The coefficient of determination (*R*^2^) of VF (LFVFS-39) and VA of the best-seeing eye (letters) and VF (LFVFS-39) and VA of the worse-seeing eye (letters) served as benchmarks. Next, a three stepwise hierarchical multiple linear regression models was constructed with VF (LFVFS-39) as dependent variable. In each model, a different retinal sensitivity measure—MS, MS cd log, or PRT—served as retinal function measure. Candidate independent variables were limited to those variables with a statistically significant F-test score from the previous analysis and the following potential confounding variables based on the literature: AMD stage,^6^ sex,^21^^,^^27^ and age.^34^ This process was iterated three times, yielding nine different regression models. The first series of three models (MS, MS cd log, and PRT resp.) were constructed using variables measured in the best-seeing eye; the second series were based on the worse-seeing eye (worse-seeing eye cohort); the third series included both eyes. For all nine models, the following assumptions were visually checked: (1) linearity, (2) multivariate normality, (3) absence of homoscedasticity, and (4) absence of multicollinearity (variance inflation factor of <10). The α was 5%. All statistical analyses were performed using IBM SPSS Statistics for Windows, version 24 (IBM Corp., Armonk, NY).

## Results

### Cohort Description

In the best-seeing eye cohort, four patients had missing data and additionally the grid was misaligned in two additional patients. This resulted in 102 patients (64 females [61%]; mean age, 71.8 ± 11.2 years) (Table 1). The mean VA was 79.6 ± 10.9 letters. In total, 40 eyes were graded as RS stage 4, of which 14 were neovascular, 21 atrophic, and 5 showed a combination. The patient stage was RS stage 4 in 84 patients.

**Table 1.** Baseline Demographics

Characteristics	Best-Seeing Eye (*n* = 102)	Worse-Seeing Eye (*n* = 84)
Age, years	71.8 ± 11.2	71.5 ± 11.5
Female,	64 (60.7)	51 (61)
VA, letters	79.6 ± 10.9	63.5 ± 20.7
Stage		
RS stage 0	3 (2.9)	0 (0)
RS stage 1	0 (0)	0 (0)
RS stage 2	29 (28.4)	10 (12)
RS stage 3	30 (29.4)	13 (15)
RS stage 4	40 (39.2)	61 (73)
Patient stage		
RS stage 0	0 (0)	0 (0)
RS stage 1	0 (0)	0 (0)
RS stage 2	9 (8.8)	10 (10)
RS stage 3	9 (8.8)	9 (11)
RS stage 4	84 (82.4)	67 (80)
RS stage 4 patient		
Neovascular	14 (13.7)	22 (26)
Atrophic	21 (20.6)	8 (10)
Combined	5 (4.9)	31 (37)

RS stage 0 corresponds with no signs of AMD at all or hard drusen <63 µm only; RS stage 1 includes soft distinct drusen (≥63 µm) only or pigmentary irregularities only, no soft drusen (≥63 µm); RS stage 2 includes soft indistinct drusen (≥125 µm) or reticular drusen only, soft distinct drusen (≥63 µm); RS stage 3 includes soft indistinct (≥125 µm) or reticular drusen with pigmentary irregularities; RS stage 4 includes atrophic or neovascular AMD.

Patient stage is defined by the worse staged eye.

Values are mean ± standard deviation or number (%).

The median of LFVFS-39 scores were 77.6 (interquartile range, 25.4; range, 22–100). Scores were affected by sex; the mean scores for males 79.9 ± 16.7 and females 72.0 ± 20.0 differed (Supplementary Fig. S1)).

A subset of 102 patients contributed data of the worse-seeing eye. Of the 18 missings, 10 patients did not perform MMP. The median VA was 20 letters (interquartile range, 47 letters) for these 10 eyes and all VA were lower than 56 letters, except for one patient. Additionally, in eight patients the grid was misaligned. The VA in the worse-seeing eye for these eight patients did not exceed 52 letters.

The worse-seeing eye cohort featured 84 patients. The mean VA was 63.5 ± 20.7 letters. Sixty-one eyes (73%) were graded as RS stage 4 (Table 1). The median LFVFS-39 scores were 82 (interquartile range, 24; range, 28–100).

### Exploratory Analysis

**Figure 2.**
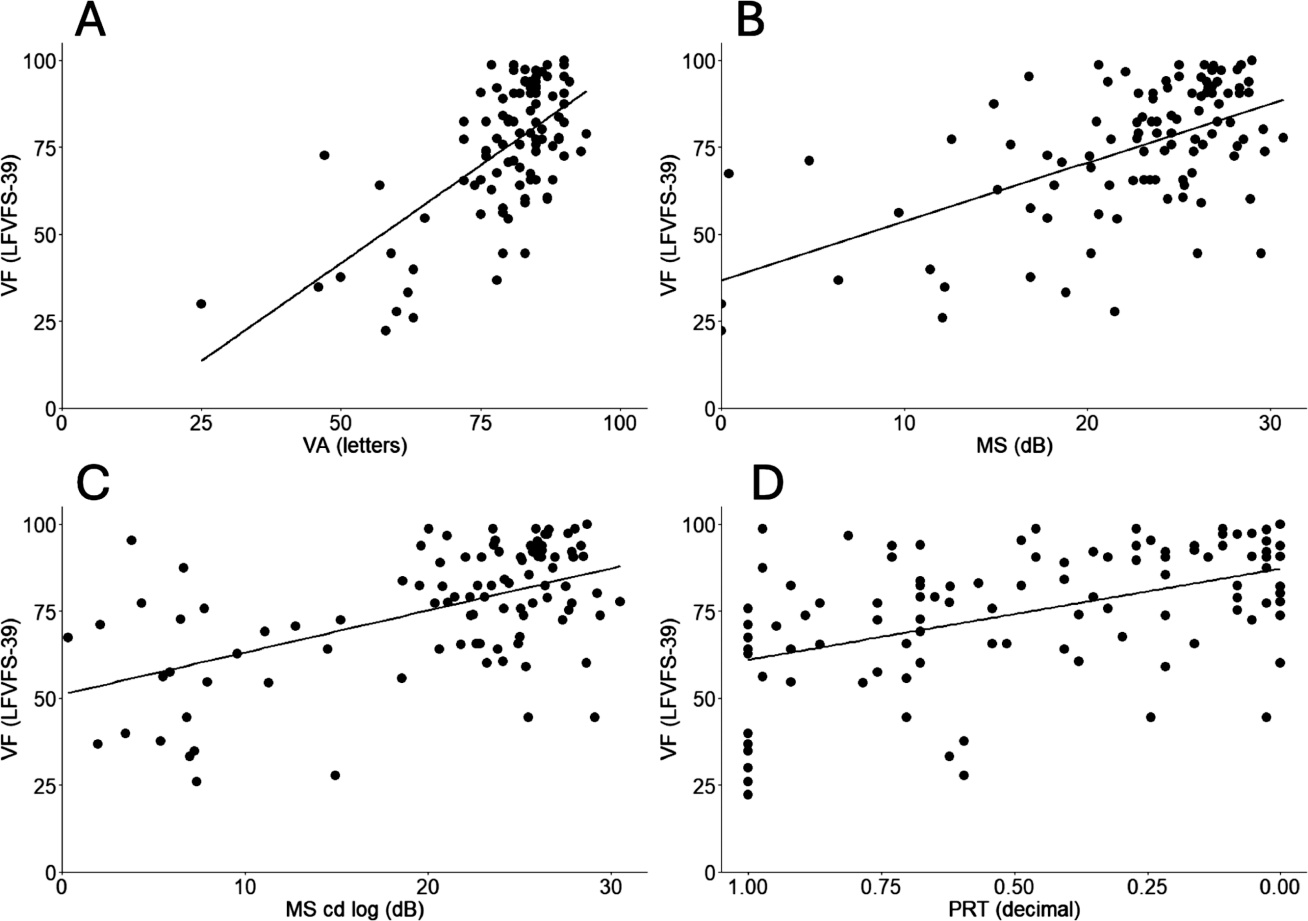
VF and retinal function measures of best-seeing eye. (**A**) Visual function (LFVFS-39) and visual acuity (letters). (**B**) Visual function (LFVFS-39) and mesopic microperimetry (MS). (**C**) Visual function (LFVFS-39) and mesopic microperimetry (MS cd log). (**D**) Visual function (LFVFS-39) mesopic microperimetry (PRT). LFVFS-39, long form visual function score; VA, visual acuity; MS, mean sensitivity; MS cd log, mean sensitivity candela log; PRT, percent reduced threshold.

A simple linear regression with each candidate variable was performed. In the best-seeing eye cohort, all F-test scores were statistically significant. VA performed best (*R*^2^ = 0.41). The retinal sensitivities were ranked MS cd log (dB) (*R*^2^ = 0.36), MS (dB) (*R*^2^ = 0.32), and PRT (decimal) (*R*^2^ = 0.23) best to worse (Table 2; Fig. 2).

**Table 2.** Simple Linear Regression With LFVFS-39

	*R* ^2^	
Variables	Best-Seeing Eye (*n* = 102)	Worse-Seeing Eye (*n* = 84)
VA (letters)	0.41^*^	0.06^*^
MS (dB)	0.32^*^	0.07^*^
MS cd log (dB)	0.36^*^	0.09^*^
PRT (decimal)	0.23^*^	0.07^*^
BCEA-63 (°) ln	0.10^*^	0.04
BCEA-95 (°) ln	0.09^*^	0.04
P1 (%)	0.16^*^	0.05^*^
P2 (%) reflect^†^ ln	0.07^*^	0.02

BCEA-63, bivariate contour ellipse area containing 63% of all fixation points BCEA-95, bivariate contour ellipse area containing 95% of all fixation points; P1, percentage of fixation within 1 degree; P2, percentage of fixation within 2 degrees.

* *P* < 0.05.

† Optimal fit was achieved by reflecting P2 values and log transforming the product.

Of the fixation metrics, P1 (%) performed best (*R*^2^ = 0.16) (Table 2; Supplementary Fig. S2). In the worse-seeing eye, the *R*^2^ of MS cd log (dB) was 0.09. All other variables performed worse (Table 2; Supplementary Fig. S3). F-scores for BCEA-63 (°), BCEA-95 (°), and P2 (%) were not statistically significant (Table 2; Supplementary Fig. S4). Of note, the BCEA-63 (°), BCEA-95 (°), and P2 (%) were transformed before all analyses. Untransformed fixation metrics in the best-seeing eye cohort are visualized in Supplementary Figure S5.

## Results

In the best seeing-cohort, VA consistently accounted for the largest portion of variance explained in LFVFS-39 (MS; *R*^2^ = 0.38, MS cd log; *R*^2^ = 0.38, PRT; *R*^2^ = 0.39) (Table 3). All retinal sensitivities significantly contributed to the proportion of total variance explained (MS; Δ*R*^2^ = 0.07, MS cd log; Δ*R*^2^ = 0.08, PRT; Δ*R*^2^ =0.03).

**Table 3.** Stepwise Hierarchical Linear Regression With LFVFS-39 of Best-Seeing Eye (*n* = 102)

	β (*P* value)		
Independent Variables	MS (dB)	MS cd log (dB)	PRT (Decimal Value)
Intercept	−18.1	−5.7	1.0
VA, letters	1.0 (<0.001)	0.9 (<0.001)	1.1 (<0.001)
Retinal sensitivity	0.9 (<0.001)	0.7 (<0.001)	−10.6 (0.02)
Sex	−8.3 (0.001)	−9.1 (0.001)	−9.0 (0.001)
Variance			
Partial adjusted *R*^2^^*^	0.45	0.46	0.40
Total adjusted *R*^2^^†^	0.49	0.50	0.45
Stepwise Δ*R*^2^			
Δ*R*^2^ VA (*P* value)	0.38 (<0.001)	0.38 (<0.001)	0.39 (<0.001)
Δ*R*^2^ Retinal sensitivity (*P* value)	0.07 (<0.001)	0.08 (<0.001)	0.03 (0.03)
Δ*R*^2^ Sex (*P* value)	0.05 (0.001)	0.06 (0.001)	0.06 (0.001)

* Partial adjusted *R*^2^ refers to a partial model consisting of a retinal sensitivity metric and VA.

† Total adjusted *R*^2^ refers to a model consisting of all independent variables.

For all three models, all assumptions were met. The variance inflation factor did not exceed 1.5. None of the other candidate variables P1 (%), P2 inv. Ln (%), BCEA-63 Ln (°), age, or RS stage were forward selected in any model. BCEA-63 Ln and BCEA-95 ln were statistically the same (*R*^2^ = 0.99); therefore, the list of candidate variables was limited to BCEA-63. Sex accounted for approximately 5% of variance in each model. Of note, RS stages 1 and 0 were collapsed to account for power.

The MS cd log model accounted for the greatest proportion of variance explained (total adjusted *R*^2^ = 0.52). The combined contribution of variance explained by VA and MS cd log was 46% (partial adjusted *R*^2^ = 0.46). The estimated βs were 0.9 (*P* < 0.001) per letter and 0.7 (*P* < 0.001) per decibel in this model (Table 2).

In the worse-seeing eye cohort (Supplementary Table S1), VA was not forward selected in the models. Only sex and retinal sensitivity were included. Total variance explained ranged from 9% to 14% (total adjusted *R*^2^) (Supplementary Table S2). All retinal sensitivities significantly contributed to the proportion of total variance explained (MS; Δ*R*^2^ = 0.07, MS cd log; Δ*R*^2^ = 0.09, PRT; Δ*R*^2^ = 0.07). All assumptions were met. None of the other candidate variables P1 (%), RS stage 4, RS stage 3, or age were included.

In the final series including both eyes, VA of the best-seeing eye was included in each model (Supplementary Table S2) PRT, regardless of best or worse-seeing eye, was not selected (Supplementary Table S3). The VA of the best-seeing eye accounted for the greatest proportion of the variance explained (MS; Δ*R*^2^ = 0.30, MS cd log; Δ*R*^2^ = 0.30, PRT; Δ*R*^2^ = 0.30). In the PRT model, the initial candidate variable list were subject to multicollinearity regarding BCEA-63 and P2 (variance inflation factor = 11.4). This model was rerun, leaving out BCEA-63. In the final models, all assumptions were met.

## Discussion

In this secondary analysis of 102 AMD patients, VA of the best-seeing eye is more strongly related to daily vision-dependent tasks (VF and LFVFS-3) than retinal sensitivity (MS, MS cd log, and PRT) or fixation metrics (BCEA-63, BCEA-95, P1, and P2) as measured via MMP. In the best-seeing eye cohort, combining VA and the best performing MMP retinal sensitivity (MS cd log) increases the proportion of variance explained from 38% (VA alone) to 46% (VA and MS cd log) (*P* < 0.001), suggesting limited added benefit of retinal sensitivity. The proportion of variance explained is important in the context of end point selection in clinical trials. Although uniocular variables cannot fully capture VF,^35^ these findings suggest that, in cases where proportional gains or losses in VA (letters) and MMP (dB) occur, VA's higher proportion of variance explained inspires greater confidence in predicting an equivalent change in VF. These findings are in line with previous work, which underscores the relative weakness of MMP compared with VA-based measures.^36^^,^^37^

MMP's relative underperformance might be explained in two ways. First, although VA and MMP measure overlapping anatomical regions—VA measures foveal function and MMP measures macular function—both are linearly associated with VF. Therefore, there might be shared variance between MMP and VA, which is absorbed by VA. Second, it could be that foveal function is more important for VF than the extrafoveal macula, reflecting cone density used for photopic vision in the human eye.^38^ Therefore, if foveal function is overall more important, and VA is a more accurate estimate of foveal function than MMP, this would explain the outperformance of VA.

Analyses with both eyes confirm the main finding that VA of the better eye outperforms MMP in the better eye. However, overall model performance was worse because MMP could not be performed in a substantial number of eyes with poor vision, resulting in fewer patients overall (*n* = 84) and a reduced range of VF scores (*R*^2^ was 39% for the best model in this series). Analyses with the worse-seeing eyes, although confounded by the absence of the strongest predictor (*R*^2^ was 14% for the best model in this series), still provides an indication of the relative predictive strengths of each MMP metric, because PRT failed to be forward selected and consistently underperformed in other analyses as well (Supplementary Tables S2 and S3).

Some notes on the different analyses of retinal sensitivity are warranted. There is ongoing experimentation with alternative analysis strategies.^5^^,^^39^^,^^40^ To our best knowledge, we are the first to perform the MS cd log transformation. In brief, according to the Weber–Fechner law, when light intensity increases exponentially, this is perceived linearly by humans.^6^ We attempted to use this to our advantage. If candela/m^2^ values are averaged before log transformation, this produces the effect of sensitizing the retinal sensitivity to scotoma formation (Fig. 1^2^). There are theoretical limits to its applicability however; in the case of extensive scotoma, this logarithmic property likely masks the presence of areas with relatively good functionality. Among retinal sensitivity parameters (MS, MS cd log, and PRT), MS cd log and MS performed comparably and PRT performed worse. Considering MS cd log's sensitivity to scotoma formation while retaining a strong relationship with VF, further confirmatory research into its utility is warranted. These suggestions likewise apply to scotopic microperimetry and may benefit ongoing studies such as MACUSTAR (Development of Novel Clinical Endpoints in Intermediate AMD), which aims to explore structure–function relationships in intermediate patients.^41^

### Strengths and Weaknesses

Although the study has strengths, including a systematic investigation of many MMP-derived variables, it also has limitations. An important limitation is the use of uniocular instead of binocular variables. In fact, in all three retinal sensitivity models of the best-seeing eye cohort, 50% of the variance remains unexplained. This factor is in part explained by the aforementioned limitation in addition to unmeasured social, cognitive, and psychological factors.^42^ Although assuredly preferable for a more accurate estimate of VF, statistical modelling on binocular vision presents power, interpretability, and feasibility challenges. To accommodate the complex interplay of both eyes, model complexity would increase, limiting overall interpretability of findings in relation to the research question. In addition, appropriate power for all relevant interactions, was, considering our sample size, not feasible.

Similarly reflecting a pragmatic solution to a complex problem, stepwise hierarchical linear regression allows for systematic and interpretable evaluation of variances in relation to overall model performance; however, it does not allow for potentially relevant interaction effects such as two eyes with highly advanced disease.^43^ Last, the NEI-VFQ–derived LFVFS-39 retains shortcomings, thus potentially contributing measurement error.^27^ In addition, VF might be intrinsically noisy. Future efforts might benefit from computer-adapted testing of VF, such as the rigorously validated EyeQ.^44^

## Conclusions

MMP is associated with daily vision-dependent tasks, but VA provides a more meaningful estimation of this same construct. These results suggest that VA provides stronger evidence of potential clinical efficacy in trials than MMP. Replications of further studies are necessary to confirm these findings.

## Supplementary Material

Supplement 1

## References

[1] Colijn JM, Buitendijk GHS, Prokofyeva E, et al. Prevalence of age-related macular degeneration in Europe: the past and the future. *Ophthalmology*. 2017; 124: 1753–1763. 28712657 10.1016/j.ophtha.2017.05.035 PMC5755466

[2] Pondorfer SG, Terheyden JH, Heinemann M, Wintergerst MWM, Holz FG, Finger RP. Association of vision-related quality of life with visual function in age-related macular degeneration. *Sci Rep*. 2019; 9: 15326. 31653904 10.1038/s41598-019-51769-7 PMC6814705

[3] Brody BL, Gamst AC, Williams RA, et al. Depression, visual acuity, comorbidity, and disability associated with age-related macular degeneration. *Ophthalmology*. 2001; 108: 1893–1900; discussion 1900–1891. 11581068 10.1016/s0161-6420(01)00754-0

[4] Csaky K, Ferris F III, Chew EY, Nair P, Cheetham JK, Duncan JL. Report from the NEI/FDA endpoints workshop on age-related macular degeneration and inherited retinal diseases. *Invest Ophthalmol Vis Sci*. 2017; 58: 3456–3463. 28702674 10.1167/iovs.17-22339 PMC5961066

[5] Yang Y, Dunbar H. Clinical perspectives and trends: microperimetry as a trial endpoint in retinal disease. *Ophthalmologica*. 2021; 244: 418–450. 33567434 10.1159/000515148 PMC8686703

[6] Pfau M, Jolly JK, Wu Z, et al. Fundus-controlled perimetry (microperimetry): application as outcome measure in clinical trials. *Prog Retin Eye Res*. 2021; 82: 100907. 33022378 10.1016/j.preteyeres.2020.100907 PMC12872260

[7] von der Emde L, Pfau M, Thiele S, et al. Mesopic and dark-adapted two-color fundus-controlled perimetry in choroidal neovascularization secondary to age-related macular degeneration. *Transl Vis Sci Technol*. 2019; 8: 7. 10.1167/tvst.8.1.7 PMC6327348 30637177

[8] Lindner M, Nadal J, Mauschitz MM, et al. Combined fundus autofluorescence and near infrared reflectance as prognostic biomarkers for visual acuity in foveal-sparing geographic atrophy. *Invest Ophthalmol Vis Sci*. 2017; 58: Bio61–bio67. 28475704 10.1167/iovs.16-21210

[9] Sleiman K, Veerappan M, Winter KP, et al. Optical coherence tomography predictors of risk for progression to non-neovascular atrophic age-related macular degeneration. *Ophthalmology*. 2017; 124: 1764–1777. 28847641 10.1016/j.ophtha.2017.06.032 PMC5768932

[10] Heier JS, Pieramici D, Chakravarthy U, et al. Visual function decline resulting from geographic atrophy: results from the Chroma and Spectri phase 3 trials. *Ophthalmol Retina*. 2020; 4: 673–688. 32199866 10.1016/j.oret.2020.01.019

[11] Csaky KG, Patel PJ, Sepah YJ, et al. Microperimetry for geographic atrophy secondary to age-related macular degeneration. *Surv Ophthalmol*. 2019; 64: 353–364. 30703401 10.1016/j.survophthal.2019.01.014 PMC6532786

[12] Hariri AH, Tepelus TC, Akil H, Nittala MG, Sadda SR. Retinal sensitivity at the junctional zone of eyes with geographic atrophy due to age-related macular degeneration. *Am J Ophthalmol*. 2016; 168: 122–128. 27189929 10.1016/j.ajo.2016.05.007

[13] Meleth AD, Mettu P, Agrón E, et al. Changes in retinal sensitivity in geographic atrophy progression as measured by microperimetry. *Invest Ophthalmol Vis Sci*. 2011; 52: 1119–1126. 20926818 10.1167/iovs.10-6075 PMC3053096

[14] Pfau M, Müller PL, von der Emde L, et al. Mesopic and dark-adapted two-color fundus-controlled perimetry in geographic atrophy secondary to age-related macular degeneration. *Retina*. 2020; 40: 169–180. 30300264 10.1097/IAE.0000000000002337

[15] Kniestedt C, Stamper RL. Visual acuity and its measurement. *Ophthalmol Clin North Am*. 2003; 16: 155–170, v. 12809155 10.1016/s0896-1549(03)00013-0

[16] Provis JM, Dubis AM, Maddess T, Carroll J. Adaptation of the central retina for high acuity vision: cones, the fovea and the avascular zone. *Prog Retin Eye Res*. 2013; 35: 63–81. 23500068 10.1016/j.preteyeres.2013.01.005 PMC3658155

[17] Wu Z, Ayton LN, Luu CD, Guymer RH. Longitudinal changes in microperimetry and low luminance visual acuity in age-related macular degeneration. *JAMA Ophthalmol*. 2015; 133: 442–448. 25632841 10.1001/jamaophthalmol.2014.5963

[18] Cocce KJ, Stinnett SS, Luhmann UFO, et al. Visual function metrics in early and intermediate dry age-related macular degeneration for use as clinical trial endpoints. *Am J Ophthalmol*. 2018; 189: 127–138. 29477964 10.1016/j.ajo.2018.02.012 PMC6043161

[19] Fleming TR, Powers JH. Biomarkers and surrogate endpoints in clinical trials. *Stat Med*. 2012; 31: 2973–2984. 22711298 10.1002/sim.5403 PMC3551627

[20] Lamoureux E, Pesudovs K. Vision-specific quality-of-life research: a need to improve the quality. *Am J Ophthalmol*. 2011; 151: 195–197. e192. 21251493 10.1016/j.ajo.2010.09.020

[21] Nickels S, Schuster AK, Elflein H, et al. Vision-related quality of life considering both eyes: results from the German population-based Gutenberg Health Study (GHS). *Health Qual Life Outcomes*. 2019; 17: 98. 31170975 10.1186/s12955-019-1158-1 PMC6554962

[22] Camparini M, Cassinari P, Ferrigno L, Macaluso C. ETDRS-Fast: implementing psychophysical adaptive methods to standardized visual acuity measurement with ETDRS charts. *Invest Ophthalmol Vis Sci*. 2001; 42: 1226–1231. 11328731

[23] Ferris FL 3rd, Kassoff A, Bresnick GH, Bailey I. New visual acuity charts for clinical research. *Am J Ophthalmol*. 1982; 94: 91–96. 7091289

[24] Early Treatment Diabetic Retinopathy Study design and baseline patient characteristics: ETDRS report number 7. *Ophthalmology*. 1991; 98: 741–756. 2062510 10.1016/s0161-6420(13)38009-9

[25] Mangione CM, Lee PP, Gutierrez PR, Spritzer K, Berry S, Hays RD. Development of the 25-item National Eye Institute Visual Function Questionnaire. *Arch Ophthalmol*. 2001; 119: 1050–1058. 11448327 10.1001/archopht.119.7.1050

[26] Han RC, Jolly JK, Xue K, MacLaren RE. Effects of pupil dilation on MAIA microperimetry. *Clin Exp Ophthalmol*. 2017; 45: 489–495. 28002873 10.1111/ceo.12907

[27] Pesudovs K, Gothwal VK, Wright T, Lamoureux EL. Remediating serious flaws in the National Eye Institute Visual Function Questionnaire. *J Cataract Refract Surg*. 2010; 36: 718–732. 20457362 10.1016/j.jcrs.2009.11.019

[28] Nickels S, Schuster AK, Singer S, et al. The National Eye Institute 25-Item Visual Function Questionnaire (NEI VFQ-25) – reference data from the German population-based Gutenberg Health Study (GHS). *Health Qual Life Outcomes*. 2017; 15: 156. 28789656 10.1186/s12955-017-0732-7 PMC5549396

[29] Prem Senthil M, Khadka J, Pesudovs K. Assessment of patient-reported outcomes in retinal diseases: a systematic review. *Surv Ophthalmol*. 2017; 62: 546–582. 28062197 10.1016/j.survophthal.2016.12.011

[30] Braithwaite T, Calvert M, Gray A, Pesudovs K, Denniston AK. The use of patient-reported outcome research in modern ophthalmology: impact on clinical trials and routine clinical practice. *Patient Relat Outcome Meas*. 2019; 10: 9–24. 30774489 10.2147/PROM.S162802 PMC6352858

[31] Morales MU, Saker S, Wilde C, et al. Reference clinical database for fixation stability metrics in normal subjects measured with the MAIA microperimeter. *Transl Vis Sci Technol*. 2016; 5: 6. 10.1167/tvst.5.6.6 PMC5113982 27867756

[32] Klein R, Davis MD, Magli YL, Segal P, Klein BE, Hubbard L. The Wisconsin age-related maculopathy grading system. *Ophthalmology*. 1991; 98: 1128–1134. 1843453 10.1016/s0161-6420(91)32186-9

[33] van Leeuwen R, Chakravarthy U, Vingerling JR, et al. Grading of age-related maculopathy for epidemiological studies: is digital imaging as good as 35-mm film? *Ophthalmology*. 2003; 110: 1540–1544. 12917169 10.1016/S0161-6420(03)00501-3

[34] Molina-Martín A, Piñero DP, Pérez-Cambrodí RJ. Normal values for microperimetry with the MAIA microperimeter: sensitivity and fixation analysis in healthy adults and children. *Eur J Ophthalmol*. 2017; 27: 607–613. 28127734 10.5301/ejo.5000930

[35] Hirneiss C . The impact of a better-seeing eye and a worse-seeing eye on vision-related quality of life. *Clin Ophthalmol*. 2014; 8: 1703–1709. 25214763 10.2147/OPTH.S64200 PMC4159393

[36] Lamoureux EL, Pallant JF, Pesudovs K, Rees G, Hassell JB, Keeffe JE. The Impact of Vision Impairment Questionnaire: an assessment of its domain structure using confirmatory factor analysis and Rasch analysis. *Invest Ophthalmol Vis Sci*. 2007; 48: 1001–1006. 17325138 10.1167/iovs.06-0361

[37] Welker SG, Pfau M, Heinemann M, Schmitz-Valckenberg S, Holz FG, Finger RP. Retest reliability of mesopic and dark-adapted microperimetry in patients with intermediate age-related macular degeneration and age-matched controls. *Invest Ophthalmol Vis Sci*. 2018; 59: AMD152–AMD159. 30372731 10.1167/iovs.18-23878

[38] Wells-Gray EM, Choi SS, Bries A, Doble N. Variation in rod and cone density from the fovea to the mid-periphery in healthy human retinas using adaptive optics scanning laser ophthalmoscopy. *Eye*. 2016; 30: 1135–1143. 27229708 10.1038/eye.2016.107 PMC4985666

[39] Hsu ST, Thompson AC, Stinnett SS, et al. Longitudinal study of visual function in dry age-related macular degeneration at 12 months. *Ophthalmol Retina*. 2019; 3: 637–648. 31060977 10.1016/j.oret.2019.03.010 PMC6684849

[40] Chang DS, Callaway NF, Steffen V, et al. Macular sensitivity endpoints in geographic atrophy: exploratory analysis of Chroma and Spectri Clinical trials. *Ophthalmol Sci*. 2024; 4: 100351. 37869030 10.1016/j.xops.2023.100351 PMC10587617

[41] Taylor LJ, Josan AS, Pfau M, Simunovic MP, Jolly JK. Scotopic microperimetry: evolution, applications and future directions. *Clin Exp Optom*. 2022; 105: 793–800. 35025727 10.1080/08164622.2021.2023477

[42] Freedman VA, Martin LG, Schoeni RF, Cornman JC. Declines in late-life disability: The role of early- and mid-life factors. *Soc Sci Med*. 2008; 66: 1588–1602. 18222580 10.1016/j.socscimed.2007.11.037 PMC2408829

[43] Baguley TS . *Serious stats: a guide to advanced statistics for the behavioral sciences*. New York: Palgrave Macmillan; 2012: xxiii, 830–xxiii, 830.

[44] Rausch-Koster TP, Luijten MAJ, Verbraak FD, van Rens G, van Nispen RMA. Calibration of the Dutch EyeQ to measure vision related quality of life in patients with exudative retinal diseases. *Transl Vis Sci Technol*. 2022; 11: 5. 10.1167/tvst.11.4.5 PMC8994198 35380613

