# Supplementary material for: Visual Acuity Provides a More Meaningful Measure of Vision-Related Functioning Than Mesopic Microperimetry in Age-Related Macular Degeneration Patients: A Cross-Sectional Study
Source: Transl Vis Sci Technol. 2026 Feb 4;15(2):5. doi: 10.1167/tvst.15.2.5 (PMC12882094; doi:10.1167/tvst.15.2.5)
Supplement: Supplement 1 [file tvst-15-2-5_s001.docx]

**Visual acuity provides a more meaningful measure of vision-related daily functioning than mesopic microperimetry in AMD patients: a cross-sectional study**

**Supplementary material**

**Supplementary Graph 1.** Distribution of LFVS-39 Values (N=102)


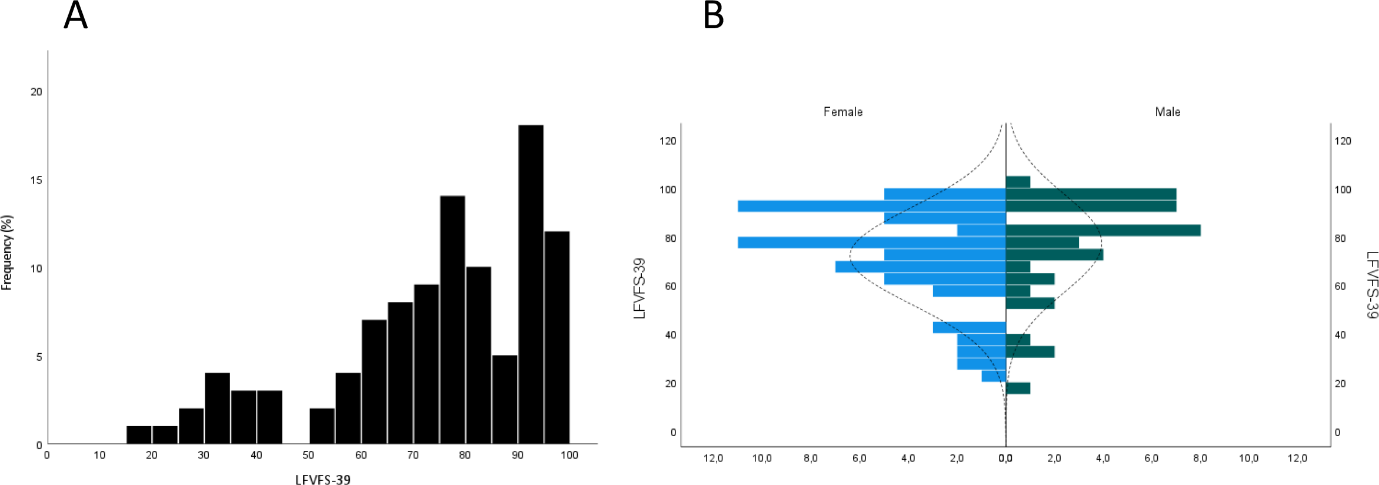


Abbreviations: LFVFS-39, long form visual function score. A: y axis: frequency of values for LFVFS-39. x axis: values for LVFS-39, value 0 corresponds to being completely unable to perform daily visual tasks. 100 corresponds to being able to perform visual daily tasks. B. Population differences men and women regarding long form visual function score or LFVFS-39.

**Supplementary Graph 2.** Visual Function and Transformed Fixation Metrics of Best-Seeing Eye


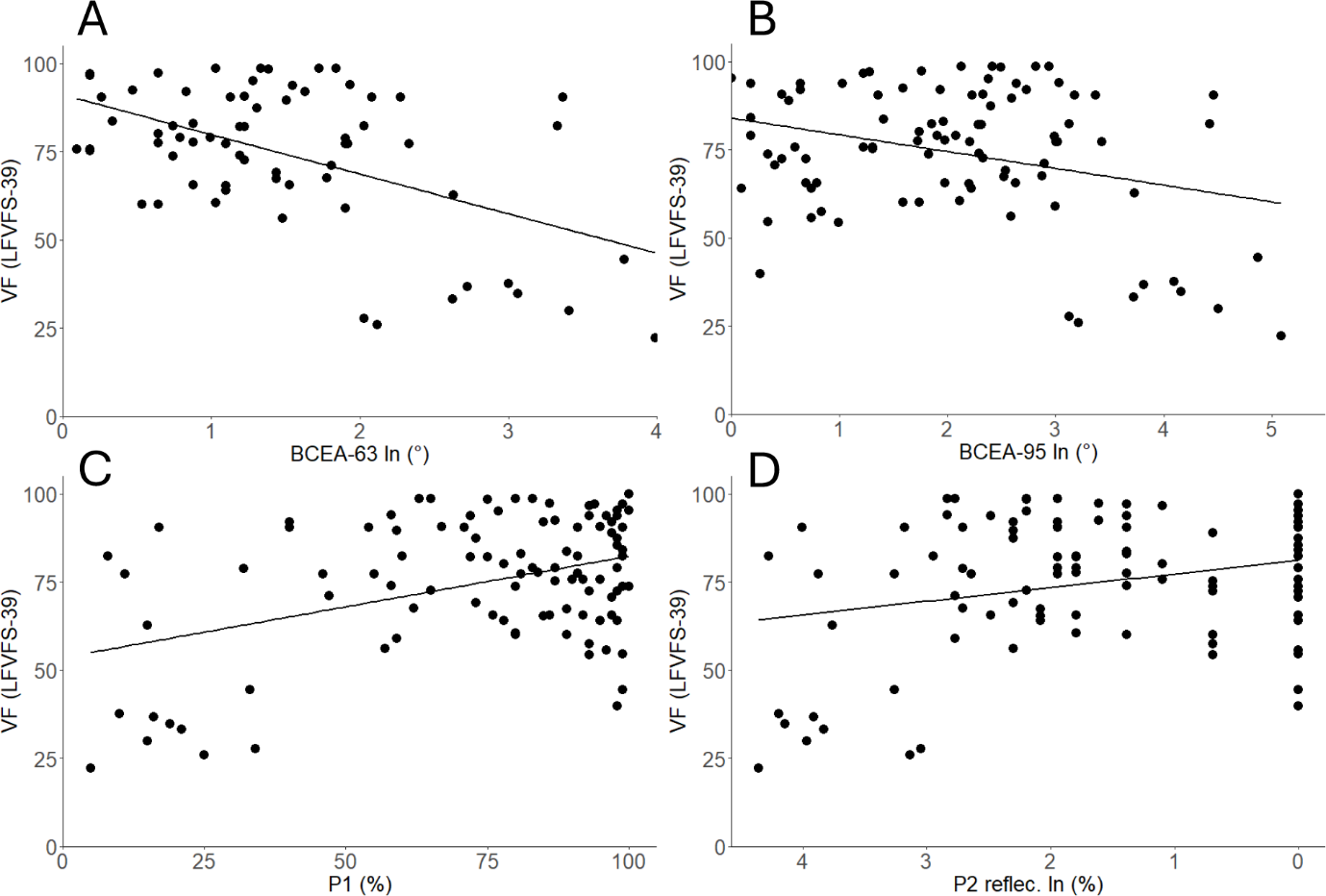


Abbreviations: LFVFS-39, long form visual function score. BCEA-63, bivariate contour ellipse area containing 63% of all fixation points. BCEA-95, bivariate contour ellipse area containing 95% of all fixation points. P1, percentage of fixation within 1 degree. P2, percentage of fixation within 2 degrees

A. LFVFS-39 and BCEA-63 natural log transformed. B. LFVFS-39 and BCEA-63 natural log transformed. C. LFVFS-39 and P1 untransformed. D. LFVFS-39 and P2, inverted and then natural log transformed.

**Supplementary Graph 3.** Visual Function and Retinal Function Measures of Worse-Seeing Eye


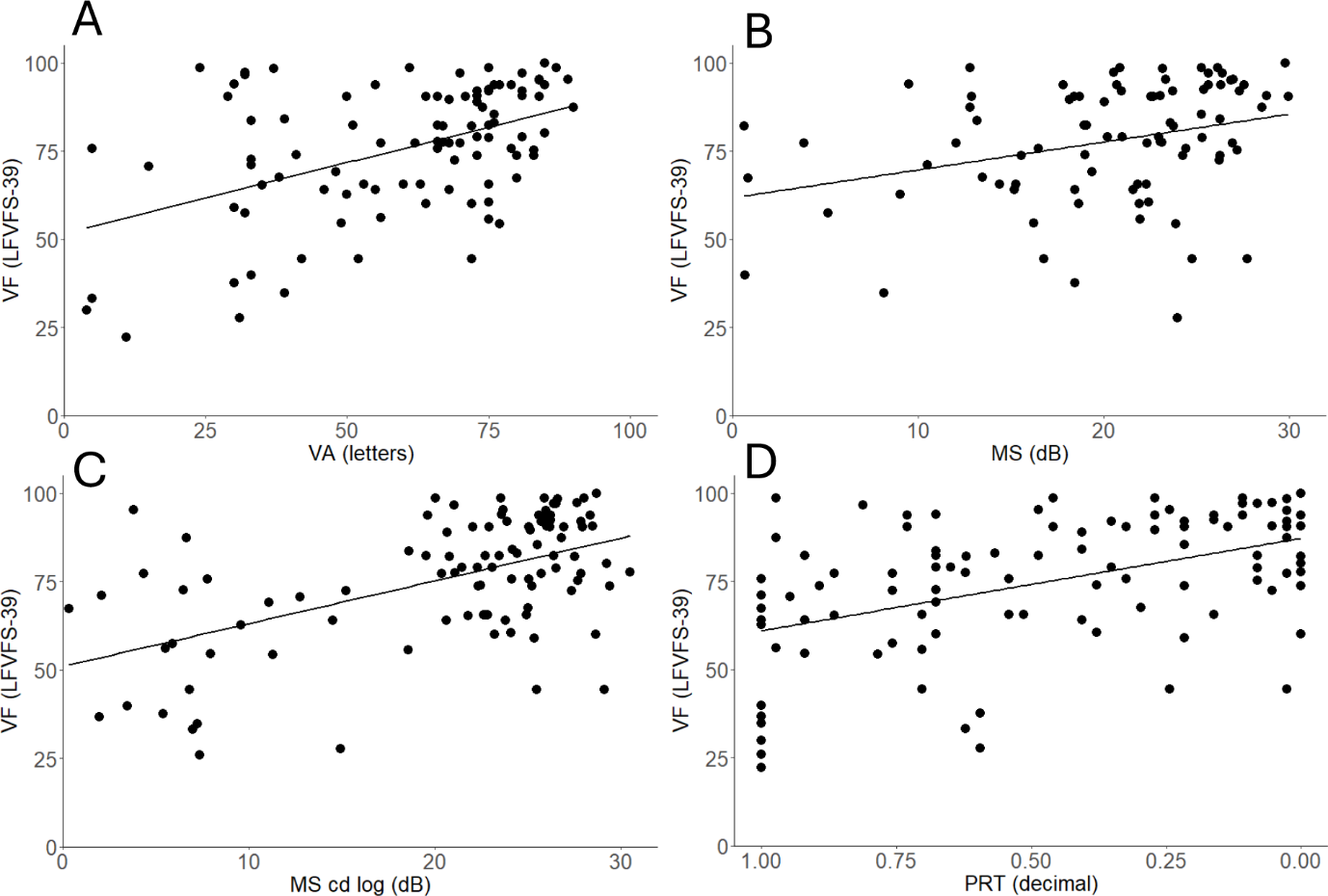


Abbreviations: LFVFS-39, long form visual function score. VA, visual acuity. MS, mean sensitivity. MS cd log, mean sensitivity candela log. PRT, percent reduced threshold.

A. LFVFS-39 and VA. B. LFVFS-39 and MS. C. LFVFS-39 and MS cd log. D. LFVFS-39 and PRT.

**Supplementary Graph 4.** Visual Function and Fixation Metrics of Worse-Seeing Eye


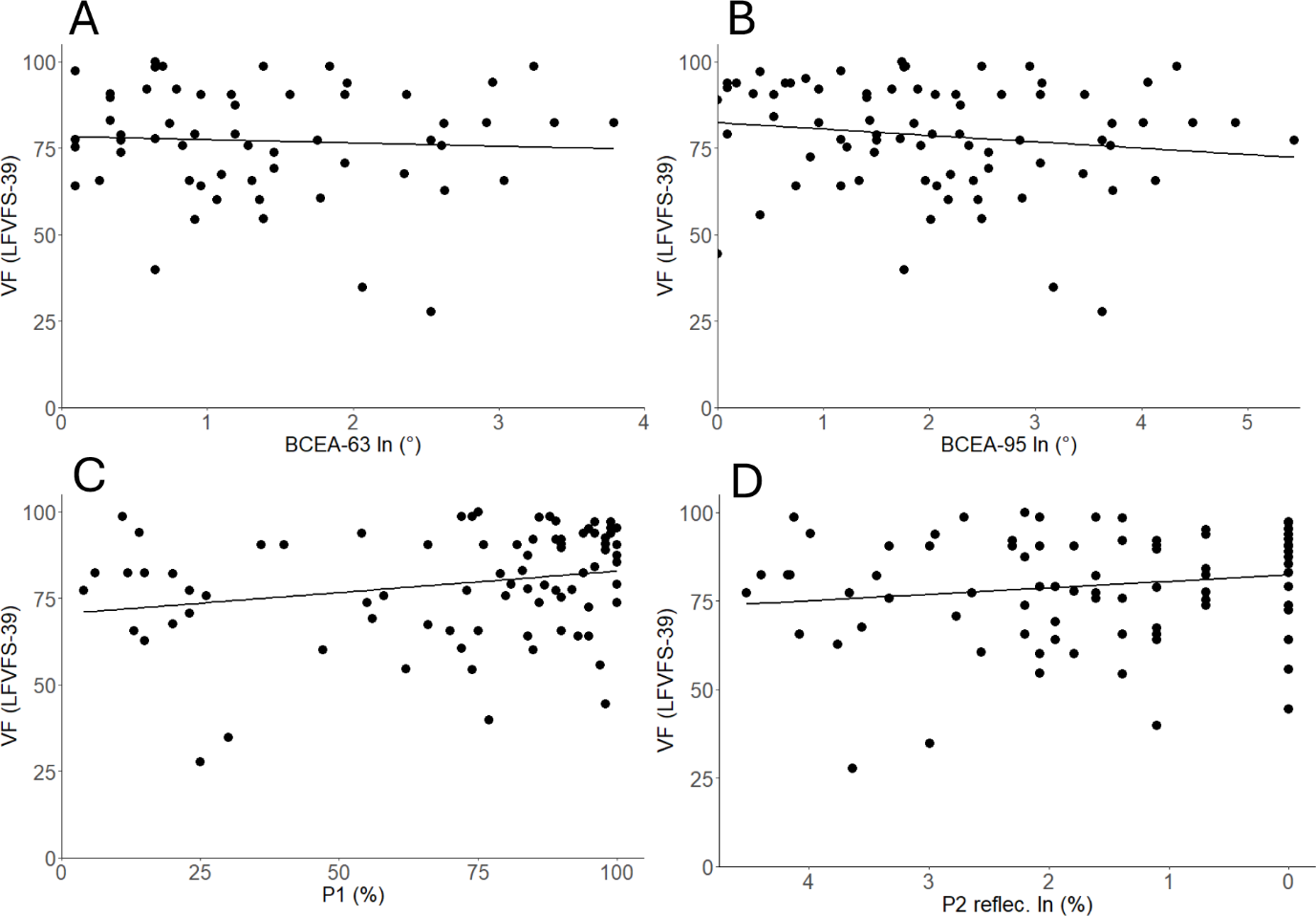


Abbreviations: LFVFS-39, long form visual function score. BCEA-63, bivariate contour ellipse area containing 63% of all fixation points. BCEA-95, bivariate contour ellipse area containing 95% of all fixation points. P1, percentage of fixation within 1 degree. P2, percentage of fixation within 2 degrees

A. LFVFS-39 and BCEA-63 natural log transformed. B. LFVFS-39 and BCEA-63 natural log transformed. C. LFVFS-39 and P1 untransformed. D. LFVFS-39 and P2, inverted and then natural log transformed.

**Supplementary Graph 5.** Visual Function and Fixation Metrics of Best-Seeing Eye

**
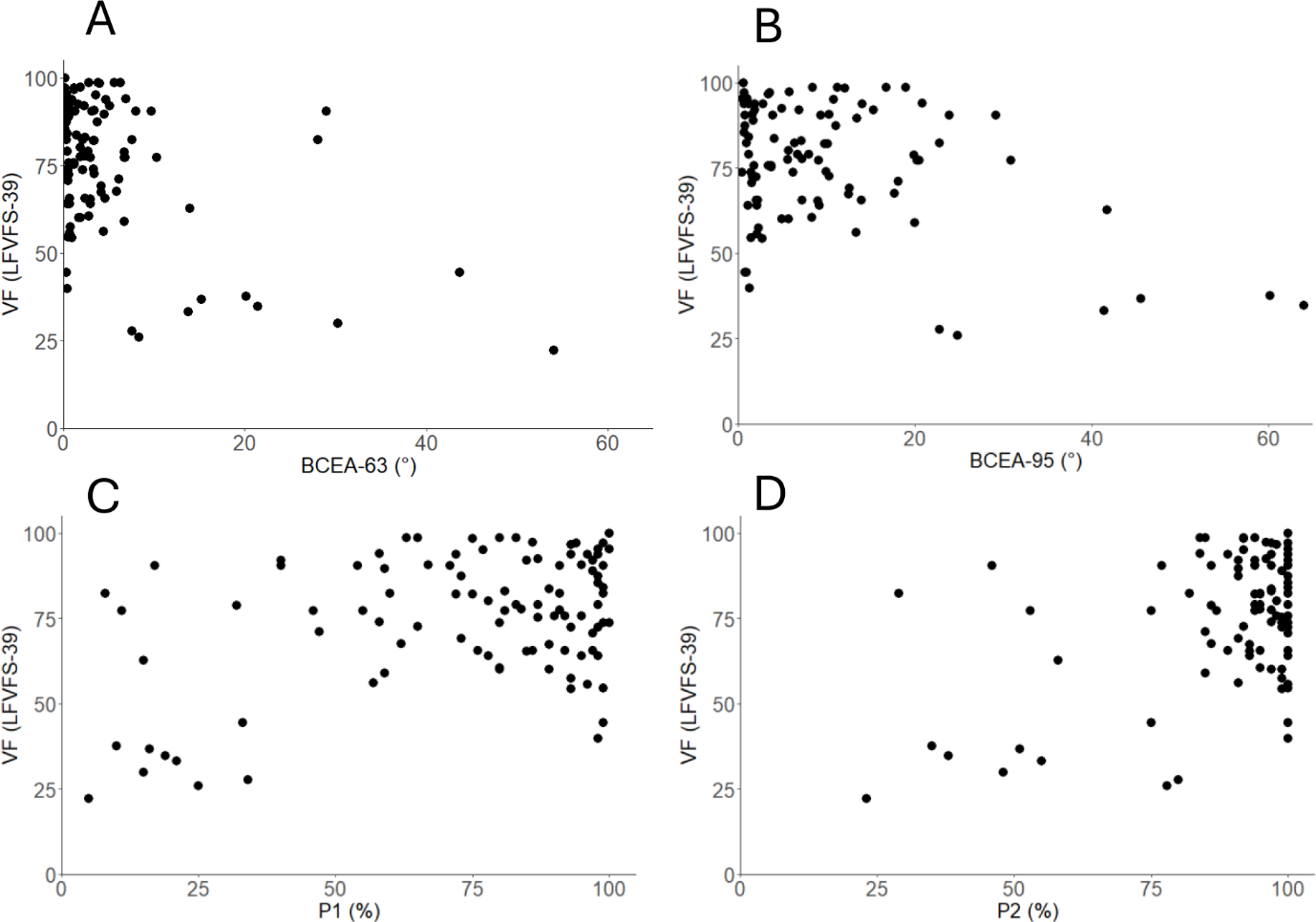
**

Abbreviations: LFVFS-39, long form visual function score. BCEA-63, bivariate contour ellipse area containing 63% of all fixation points. BCEA-95, bivariate contour ellipse area containing 95% of all fixation points. P1, percentage of fixation within 1 degree. P2, percentage of fixation within 2 degrees

A. LFVFS-39 and BCEA-63. B. LFVFS-39 and BCEA-63. C. LFVFS-39 and P1. D. LFVFS-39 and P2.

**Supplementary Table 1.** Stepwise Hierarchical Linear Regression with LFVFS-39 of Worse-Seeing Eye (n=84)

|  | **MS (dB)** | **MS cd log (dB)** | **PRT (decimal)** |
| --- | --- | --- | --- |
| Independent variables | $\beta$(*P* value) | $\beta$(*P* value) | $\beta$(*P* value) |
| Intercept | 71.4 | 73.8 | 92.3 |
| Retinal sensitivity | .6(.001) | .6(.001) | -14(.01) |
| Sex | .-7.5(.03) | -7.3(.03) | -6.7(.05) |
| Variance |  |  |  |
| Total adjusted R^2^*^*^* | .13 | .14 | .09 |
| Stepwise $\Delta$R^2^ |  |  |  |
| $\Delta$R^2^ Retinal sensitivity (*P* value) | .07(.01) | .09(.01) | .07(.02) |
| $\Delta$R^2^ Sex (*P* value) | .05(.03) | .05(.03) | .04(.05) |

Abbreviations: MS, mean sensitivity, MS cd log, mean sensitivity candela log, PRT, percent reduced threshold,

***** Total adjusted R^2^ refers to a model consisting of all independent variables.

**Supplementary Table 2.** Stepwise Hierarchical Linear Regression with LFVFS-39 Best and Worse Eye (n=84)

|  | **MS WE (dB)** | **MS cd log BE (dB)** | | **PRT (decimal value)** | |
| --- | --- | --- | --- | --- | --- |
| Independent variables | $\beta$(*P* value) | | $\beta$(*P* value) | | $\beta$(*P* value) |
| Intercept | -11.2 | -6.2 | | -24.5 | |
| VA BE, letters | 1.1 (<.001) | .9(<.001) | | 1.1 (<.001) | |
| Sex | -8.6(.001) | -8.1(01) | | -8.5(.01) | |
| Retinal sensitivity | .40(.03) | .6(.01 | |  | |
| P1 WE (decimal) |  |  | | .2(.01) | |
| P2 reflec†. Ln BE (decimal) |  |  | | 9.9(.01) | |
|  |  |  | |  | |
| Variance |  |  | |  | |
| Total adjusted R^2^* | .37 | .39 | | .41 | |
| Stepwise $\Delta$R^2^ |  |  | |  | |
| $\Delta$R^2^ VA BE (*P* value) | .30 (<.001) | .30 (<.001) | | .30 (<.001) | |
| $\Delta$R^2^ Sex (*P* value) | .05(.01) | .05(.01) | | .05(.01) | |
| $\Delta$R^2^ Retinal sensitivity (*P* value) | .04(.03 | .06(.01) | |  | |
| $\Delta$R^2^ P1 WE (decimal) |  |  | | .03(.05) | |
| $\Delta$R^2^ P2 reflec†. Ln BE (decimal) |  |  | | .04(.02) | |

Abbreviations: MS, mean sensitivity. MS cd log, mean sensitivity candela log. PRT, percent reduced threshold. BE, best seeing eye. WE, worse seeing eye. BCEA-63, bivariate contour ellipse area containing 63% of all fixation points. BCEA-95, bivariate contour ellipse area containing 95% of all fixation points. P1, percentage of fixation within 1 degree. P2, percentage of fixation within 2 degrees.

***** Total adjusted R^2^ refers to a model consisting of all independent variables.
